# Standards of care in mesothelioma treatment

**DOI:** 10.1038/s41416-020-01078-y

**Published:** 2020-09-22

**Authors:** Susan V. Harden, Liz Darlison, Paul Beckett, Anna C. Bibby

**Affiliations:** 1 Cambridge University Hospitals NHS Foundation Trust, Cambridge, UK; 2 University Hospitals of Leicester, Leicester, UK; 3 University Hospitals of Derby and Burton NHS Foundation Trust, Derby, UK; 4 Academic Respiratory Unit, Bristol University, Bristol, UK

**Keywords:** Mesothelioma, Health policy

## Abstract

The UK has the highest incidence of mesothelioma in the world, but services vary across the country partly due to uneven geographical distribution of cases. The Mesothelioma UK-funded national organisational audit has highlighted challenges in accessing diagnostic procedures such as thoracoscopy, as well as identifying examples of best practice, including access to clinical trials and specialist therapeutic procedures. To ensure equitable and optimal patient care, cancer alliances should have established referral pathways to specialist multidisciplinary team (MDT) services for discussion of all mesothelioma patients.

## Main

The UK has the highest incidence of mesothelioma worldwide, with ~2700 cases diagnosed per year. The majority of newly diagnosed people are aged 75 and above, and over 80% are men. At least 90% of cases are preventable, caused by workplace asbestos exposure decades previously.^1^ Despite these figures, mesothelioma accounts for <1% of all new cancer cases in the UK, with the number of new cases diagnosed per year in any one hospital ranging from 1 to 40.^2^ To ensure that access to treatment and care for mesothelioma patients is equitable across the country, regardless of the number of cases seen in each centre, it is important to have established regional referral pathways that facilitate access to specialist services that may not be available locally. With over 95% of mesothelioma arising in the pleural cavity, the 2013 NHS England service specifications contract recommended that “malignant mesothelioma services should be provided by a combination of lung cancer multidisciplinary teams and specialist mesothelioma multidisciplinary teams working in collaboration”. It stated that “local and regional incidence of the disease should be taken into account to ensure proper population coverage”.^3^

Treatment options remain limited for mesothelioma and, indeed, active oncologic treatment may not be appropriate for a proportion of this patient cohort. However, for fit patients of good performance status, standard first line systemic anti-cancer therapy (SACT) of pemetrexed/cisplatin doublet has not changed in the UK for over a decade. More recently, the addition of bevacizumab, where available, has been shown to be beneficial.^4,5^ There is currently no National Institute for Health and Care Excellence (NICE) approved second-line therapy. Clinical trials of new therapies (including immunotherapy) are ongoing and, with the highest incidence of cases worldwide, the UK is best placed to deliver high-quality research in this area. Indeed the UK has successfully run the only randomised trial investigating the role of radical surgery in pleural mesothelioma,^6^ practice-changing randomised trials demonstrating prophylactic irradiation of intervention sites was not necessary.^7,8^ and the University of Glasgow currently lead the PREDICT-Meso international collaborative research initiative, part-funded by Cancer Research UK.

Quality healthcare is not just about active oncologic treatment, however, for optimal patient-centred outcomes, diagnostic efficiency, pleural effusion management and access to specialist supportive, palliative and end of life care are also important.

In recent years, the charity Mesothelioma UK has funded the National Mesothelioma Audit, run by the Royal College of Physicians, to report clinical outcomes and set quality indicators and recommendations for mesothelioma care.^2^ This biennial audit gives a national and regional picture of the numbers of mesothelioma patients being diagnosed, of the treatments they receive and of their survival outcomes. Since the publication of the British Thoracic Society 2018 Mesothelioma Guidelines,^9^ the audit has evaluated care across the UK against the standards set out in this document. Although this is a useful tool for highlighting variation around the country, it does not provide detail on how services meet guideline recommendations, nor does it collect data on diagnostic services or distinguish between local thoracic versus specialist mesothelioma multidisciplinary team (MDT) discussion. The recently published national mesothelioma organisational audit was commissioned to obtain this information by collecting data on the structure of mesothelioma services across the country.^10^

All known thoracic cancer MDTs across England, Wales, Scotland and Northern Ireland were invited to take part in the organisational audit. Questions were designed to obtain an accurate picture of access to diagnostic, treatment and nursing services for mesothelioma, and to identify those teams considered specialist mesothelioma MDTs (defined as managing over 25 new cases per year).^9^ An additional survey was completed by all 17 identified specialist mesothelioma MDTs as well as the national peritoneal mesothelioma MDT at Basingstoke (~4% of UK mesothelioma cases arise in the peritoneal cavity).^2^

At a local level, the biggest challenges identified by the organisational audit were access to local anaesthetic thoracoscopy and insertion of intra-pleural catheters. These interventions are important for obtaining a tissue diagnosis and as an option for managing breathlessness in patients with symptomatic pleural effusions.

Compared with local teams, patients referred to specialist mesothelioma MDTs were more likely to have clinical stage and histological subtype recorded; important factors in assessing prognosis and clinical trial eligibility. Patients discussed at specialist mesothelioma MDTs also had increased access to on-site support from mesothelioma specialist nurses and opportunities to participate in clinical trials. All specialist MDTs had direct access to SACT and radiotherapy services, as well as established pathways for onward referral of appropriate patients to the limited number of UK centres offering radical debulking pleural surgery, cytoreductive peritoneal surgery and palliative interventions such as percutaneous cervical cordotomy.

In addition to these examples of best practice, the organisational audit identified several areas of variation between specialist MDTs. There was a range in the number of mesothelioma cases discussed, whether the mesothelioma MDT was held entirely separately or as a section within a general thoracic MDT and the extent of on-site specialist mesothelioma services offered. Specifically, mesothelioma specialist nurses and palliative care team members were not universally present in specialist mesothelioma MDT meetings. Additionally, the availability of teleconferencing facilities was considered beneficial to give immediate feedback to referring teams but was underutilised by specialist MDTs.

Recommendations from this first organisational audit of UK mesothelioma services emphasise the importance of clear referral pathways to a specialist mesothelioma MDT. Each cancer alliance or region should have designated processes via which all patients can be discussed at specialist MDT and are able to access services that are not locally available. Specialist mesothelioma MDTs, both current and future, should ensure that they are able to offer patients access to all elements of guideline-recommended treatment and the opportunity to participate in clinical trials, as well as being able to provide rapid feedback to referring teams.

## Data Availability

All data within this article is available through the cited references.
